# Circ 0020938 inhibits hair follicle stem cells proliferation via the miR-142-5p/*DSG4* axis in cashmere goats

**DOI:** 10.1186/s12864-025-11642-6

**Published:** 2025-05-19

**Authors:** Jiamian Du, Menghua Sui, Zhihao Song, Shuangshuang Liang, Yujie Zheng, Xin Wang

**Affiliations:** Key Laboratory of Animal Genetics, Breeding and Reproduction of Shaanxi Province, College of Animal Science and Technology, Northwest A&F University, Yangling, 712100 China

**Keywords:** Circ 0020938, miR-142-5p, *DSG4*, HFSCs

## Abstract

**Background:**

Shaanbei white cashmere goat is an excellent cashmere goat breed, and its market favored cashmere from the secondary hair follicles. Hair follicles mature around birth and each hair follicle repeatedly undergoes a growth cycle that comprises three distinct stages: anagen, catagen and telogen. Understanding the molecular mechanisms controlling cyclic hair follicle changes is essential for optimizing hair follicle function and improving cashmere production.

**Methods:**

The circRNA expression profile in the hair follicle cycle was constructed and differentially expressed circRNAs were identified, with particular focus on circ 0020938, which was highly expressed during anagen. The functional assays were performed to assess the effect of circ 0020938 on hair follicle stem cells (HFSCs) proliferation. Competing endogenous RNA (ceRNA) network was constructed to investigate the interaction between circ 0020938, miR-142-5p, and *DSG4*. Rescue experiment was conducted to validate the impact of circ 0020938 on HFSCs proliferation and *DSG4* expression.

**Results:**

We found that circ 0020938 inhibited HFSCs proliferation. Further analysis revealed that circ 0020938 acted as a sponge for miR-142-5p, alleviating the repression of *DSG4*. Additionally, we confirmed that *DSG4* inhibited HFSCs proliferation, suggesting that it play a key role in regulating the balance between proliferation and differentiation during the hair follicle cycle. Rescue experiments showed that the inhibition of HFSCs proliferation by circ 0020938 was partially reversed by miR-142-5p.

**Conclusion:**

Our study provides novel insights into the regulatory role of circRNA in HFSCs proliferation during the hair follicle cycle. The results demonstrate that circ 0020938 acts as a miRNA sponge and inhibits HFSCs proliferation through the miR-142-5p/*DSG4* axis, thereby contributing to the proper progression of the hair follicle cycle.

**Supplementary Information:**

The online version contains supplementary material available at 10.1186/s12864-025-11642-6.

## Introduction

The Shaanbei white cashmere goat, distributed in the north region of Shaanxi province, China, is an important breed primarily known for its cashmere production, with dual purposes of both cashmere and meat [1, 2]. This breed produces high-quality cashmere, which is highly favored in the home and abroad market [3]. The secondary hair follicles of cashmere goat are the organs responsible for cashmere production, and their most notable feature is their ability to self-renew and undergo cyclical changes [4–6]. After birth, mature hair follicles are periodically regenerated by spontaneously undergoing repetitive cycles of anagen, catagen, and telogen [6]. The Shaanbei white cashmere goat experiences a cyclical transition from telogen stage to anagen stage each year, a process that significantly affects cashmere yield [7]. Therefore, revealing the molecular mechanisms behind the hair follicle cycle in cashmere goats is of great importance for improving cashmere production.

The hair follicle cycle of cashmere goats depends on unique follicular epithelial and mesenchymal components and their interactions [8]. The periodic growth of hair follicles is driven by hair follicle stem cells (HFSCs) [9]. In the end of telogen, HFSCs adjacent to the dermal papilla cells in the bulge region are activated [10]. When the quiescent signals from the inner bulge and other HFSCs niches are overwhelmed by the combination of BMP inhibition and Wnt activation signals, the hair follicles begin to enter the anagen stage [11]. Progenitor cells located in the hair matrix proliferate rapidly, generating transit-amplifying cells, which then give rise to downstream hair follicle lineages, including hair shafts, companion layers, and the inner root sheath [12]. During the entire anagen stage, dermal papilla cells regulate the lineage selection of transit-amplifying cells [12].

Recent surges in research have demonstrated that non-coding RNAs (ncRNAs) play important regulatory roles in several key biological processes that influence development, differentiation, and metabolism [13, 14]. CircRNAs, as emerging ncRNAs, are structurally different from lncRNAs and their 3’ and 5’ ends are covalently linked [15]. Previously, the expression profile of circRNAs in hair follicle of sheep, Inner Mongolian cashmere goats, Yangtze River Delta white goat, and Liaoning cashmere goats has been identified [16–20]. Moreover, there is evidence that circRNAs play an important regulatory role as miRNA sponges during the hair follicle cycle, for example, circRNA-1967 acts as a miR-93-3p sponge, which in turn enhances *LEF1* expression and is involved in the differentiation of goat secondary hair follicle stem cells (SHF-SCs) into hair follicle lineages [21]. Circ0026326 is involved in the regulation of hair follicle cycle as a miR-320-3p sponge [22]. However, the expression profile of circRNAs during the hair follicle cycle in Shaanbei white cashmere goat remains unknown, and circRNAs regulating this process are urgently need to be identified and validated.

Therefore, in this study, we used full-transcriptome sequencing data from the skin of cashmere goats in both anagen and telogen stages to construct a circRNA expression profile and identified a differentially expressed circRNA named circ 0020938. Subsequently, we explored the function and potential regulatory mechanisms of circ 0020938 on HFSCs. The results of this study contribute to the current theoretical knowledge regarding the regulatory role of circRNAs in hair follicle cycling.

## Results

### Identification of differentially expressed circrnas in goat hair follicle anagen and telogen stages

**Fig. 1:**
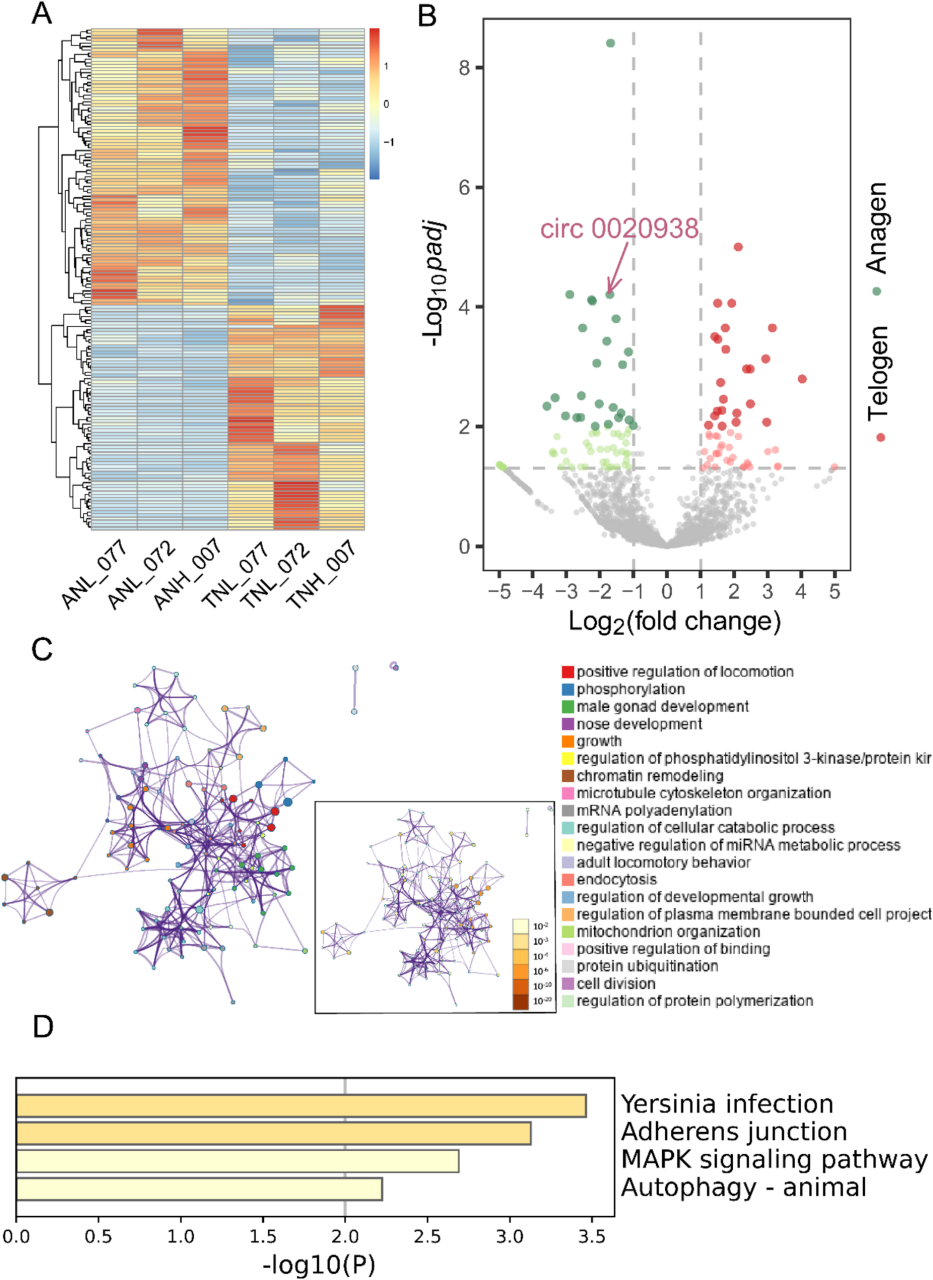
Differentially expressed circRNAs were identified in the anagen and telogen stages of hair follicles. **A** Heatmap of differentially expressed circRNAs during the anagen and telogen stages of hair follicles. **B** Volcano plot of differentially expressed circRNAs with arrows indicating circ 0020938. **C** The GO analysis of the parental genes of differentially expressed circRNAs highlights the top 20 GO terms in the main chart, while the inset displays the significance of each term. **D** KEGG analysis results for parental genes of differentially expressed circRNAs

We reanalyzed RNA-seq datasets of the skin samples of anagen and telogen stages of the cashmere goat in our laboratory (six transcriptomic datasets from the anagen and telogen stages of Shaanbei white cashmere goats) with the current analysis method, and the circRNA expression profile of the goat hair follicle at anagen and telogen stages was constructed using find_circ [23] and CIRI [24] software for joint analysis. A total of 9,096 circRNAs were identified from the skin transcriptome sequencing data of goats in both the anagen and telogen stages. The expression levels of circRNAs were quantified and normalized using TPM values. Differential expression analysis was performed with the DESeq2 [25] software, resulting in 154 differentially expressed circRNAs as potential candidates for regulating the hair follicle cycle (Fig. 1A). Among them, 85 circRNAs were upregulated in the anagen stage, while 69 circRNAs were upregulated in the telogen stage. Then circRNA 0020938 attracted our attention due to its significant differential expression (Fig. 1B).

The parental genes of differentially expressed circRNAs were used for enrichment analysis. Gene ontology (GO) and kyoto encyclopedia of genes and genomes (KEGG) pathway enrichment analyses were conducted on the parental genes of the differentially expressed circRNAs. According to the GO enrichment results, significantly enriched biological processes mainly included positive regulation of locomotion, phosphorylation and male gonad development (Fig. 1C). In the KEGG analysis results, differentially expressed circRNAs were enriched to the MAPK signaling pathway, which is essential for hair follicle cycle regulation and maintenance of hair follicle stem cells (Fig. 1D).

### Characterization of circ 0020938

**Fig. 2:**
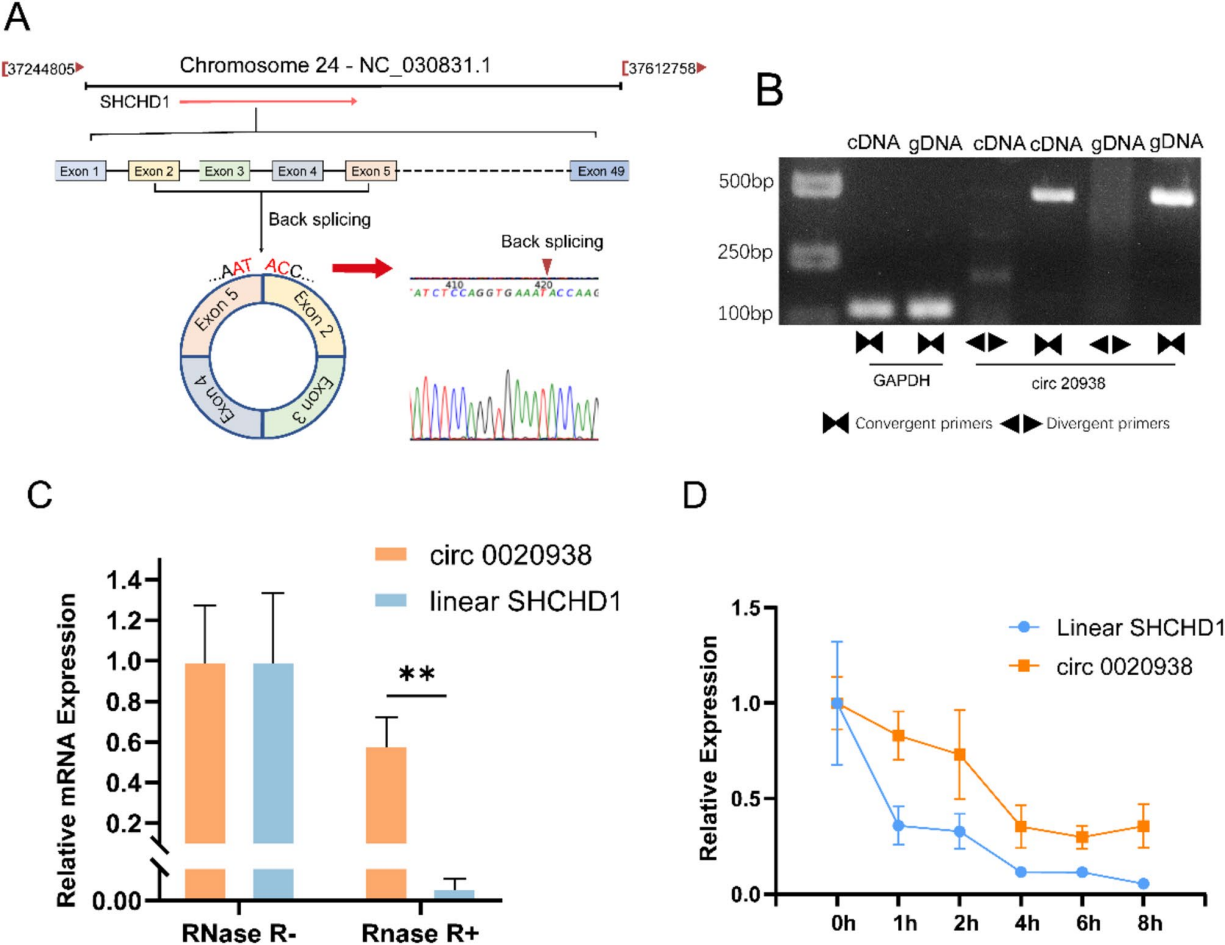
Characterization of circ 0020938. **A** The location of circ 0020938 on the goat genome and its back splice site were confirmed by sanger sequencing. **B** PCR and agarose gel electrophoresis analysis of divergent and convergent primers in cDNA and gDNA. **C** The stability of circ 0020938 and *SHCHD1* mRNA was measured after RNase R treatment. **D** RT-qPCR analysis of circ 0020938 and *SHCHD1* in hair follicle stem cells following actinomycin D treatment. **P < *0.01*

To minimize the possibility of false positives, circ 0020938, which exhibited the most abundant expression, was selected for further investigation. Circ 0020938 originates from the *SHCHD1* gene on chromosome 24 of goat. It is formed by head-to-tail splicing between exon 2 and exon 3 of the *SHCHD1* gene (Fig. 2A). To confirm the splicing junction, divergent primers flanking the circRNA splicing site were used for amplification, and the products were verified by Sanger sequencing (Fig. 2A). The use of convergent primers enabled the amplification of target sequence from both cDNA and genomic DNA (gDNA) templates, whereas the convergent primers could only amplify the target sequence from cDNA, confirming the circRNA’s unique circular structure (Fig. 2B). Moreover, RNase R and actinomycin D assays demonstrated that circRNA 0020938 exhibited greater stability compared to the linear mRNA transcribed from *SHCHD1* (Fig. 2C and D). These findings further support the conclusion that circ 0020938 possesses a stable circular structure.

### Circ 0020938 suppresses hair follicle stem cells proliferation

**Fig. 3:**
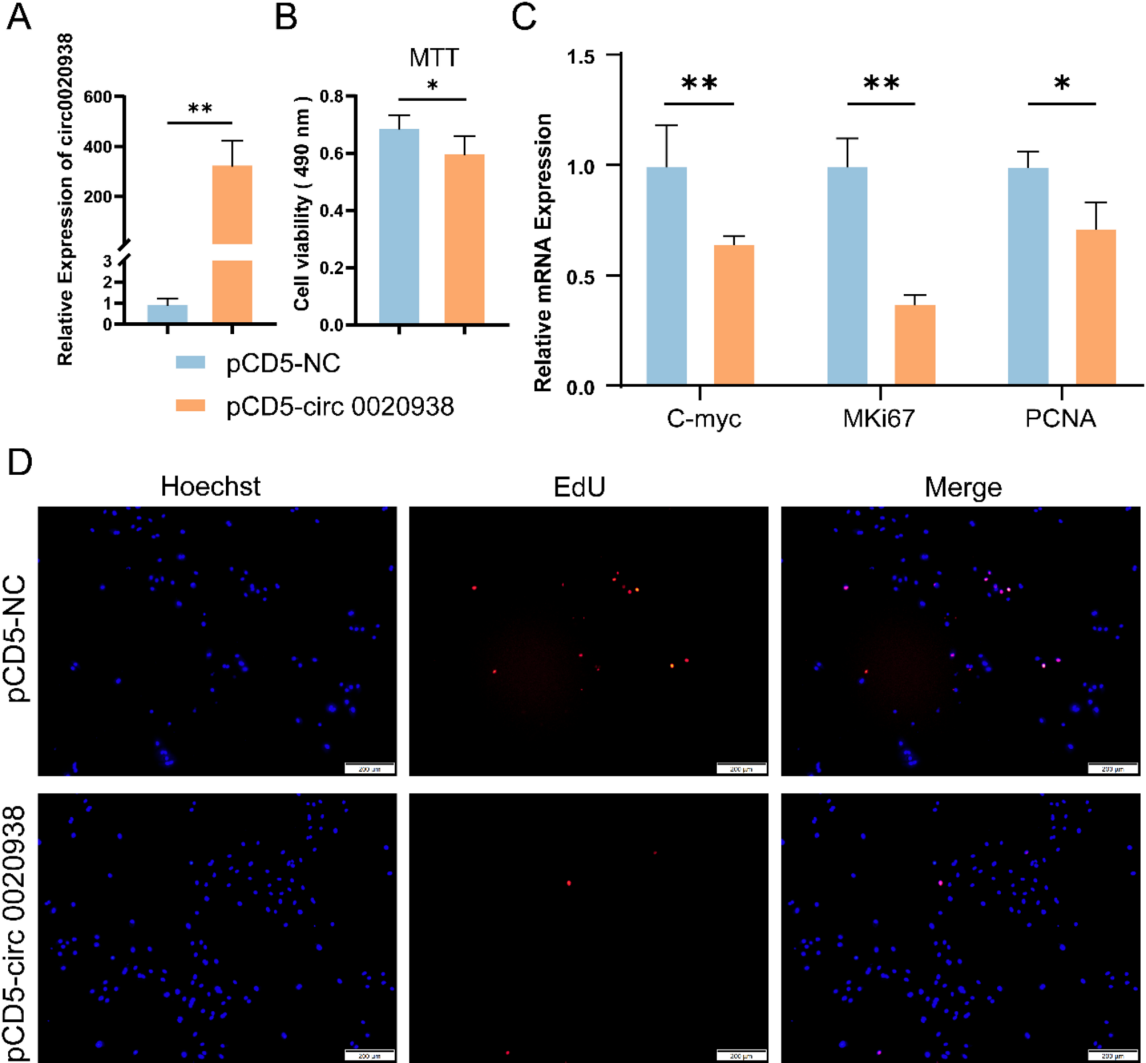
Circ 0020938 Inhibits Hair Follicle Stem Cells Proliferation. **A** RT-qPCR analysis in goat hair follicle stem cells 48 h after electrotransfection with circ 0020938 pCD5-circ 0020938. **B** MTT assay was performed to detect the effect of circ 0020938 on the viability of HFSCs after overexpression of circ 0020938. **C** RT-qPCR was used to detect the expression of proliferation-related genes (*C-myc*, *MKi67*, *PCNA*) after overexpression of circ 0020938. **D** EdU assay was used to detect the effect of circ 0020938 on the proliferation of HFSCs after the same treatment. Red represents EdU staining and blue represents cell nuclei stained with Hoechst 33,342. Data are presented from three independent experiments as mean ± SD, * *P* < 0.05, ** *P* < 0.01. Scale bar, 200 μm

To explore the functional role of circ 0020938, a circ 0020938 overexpression vector named pCD5-circ 0020938 was constructed and transfected into HFSCs via electroporation. RT-qPCR analysis showed that the expression level of circ 0020938 was significantly increased compared to the control group (*P* < 0.01) (Fig. 3A). Furthermore, MTT and EdU assays were performed to assess HFSCs proliferation on 48 h after pCD5-circ 0020938 transfection. The results indicated a significant decrease in cell viability in the pCD5-circ 0020938 group compared to the control group (*P* < 0.05) (Fig. 3B and D). Moreover, RT-qPCR analysis revealed that the expression levels of cell proliferation markers, including *C-myc*, *MKi67* and *PCNA* were significantly downregulated in the pCD5-circ 0020938 group (*P* < 0.01, *P* < 0.05), suggesting that circ 0020938 plays a role in inhibiting HFSCs proliferation (Fig. 3C).

### Circ 0020938 functions as a sponge for MiR-142-5p

**Fig. 4:**
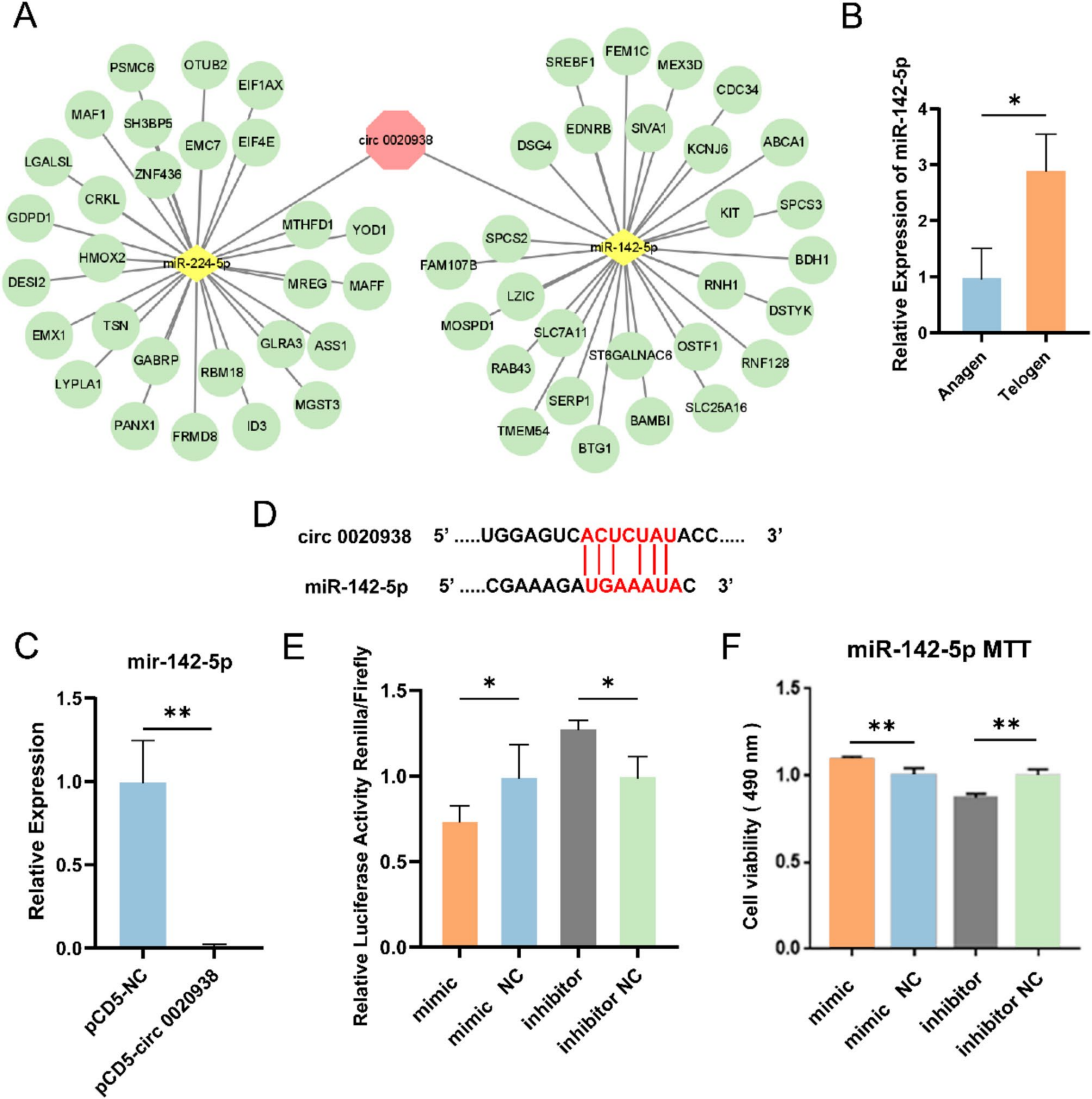
Circ 0020938 functions as a sponge for miR-142-5p. **A** CircRNAs-miRNAs-mRNAs networks. **B** The expression level of mir-142-5p in the anagen and telogen. **C** RT-qPCR of miR-142-5p expression after transfection with pCD5-NC and pCD5-circ 0020938. **D** Predicted binding sites of circ 0020938 and miR-142-5p. **E** The relative luciferase activities were detected in HEK293T cells after co-transfection with psiCHECK-circ0020938 and mimic or inhibitor. **F** MTT analysis of HFSCs proliferation. Data are presented from three independent experiments as mean ± SD, * *P* < 0.05, ** *P* < 0.01

According to bioinformatics analysis using miRanda and TargetScan, the analysis result showed that miR-142-5p and miR-224-5p had the potential to bind circ 0020938, and in addition, miRNA target genes were selected by combining differential expression analysis during the telogen and anagen (Fig. 4A). Since miR-142-5p has been implicated in various skin diseases such as alopecia and melanoma [26], we focused on its potential role in the hair follicle cycle. Subsequently, the expression of miR-142-5p in telogen was significantly higher than that in anagen using RT-qPCR, which was opposite to the expression of circ 0020938 (*P* < 0.05) (Fig. 4B). Moreover, miR-142-5p expression was significantly lower after the overexpression of circ 0020938 than that in controls (*P* < 0.01) (Fig. 4C). The dual-luciferase reporter assay was used to verify the binding of miR-142-5p to circ 0020938. Figure 4D showed the binding site of miR-142-5p to circ 0020938. Compared with the corresponding controls, the co-transfection of psiCHECK-circ0020938 with miR-142-5p mimic significantly reduced the renilla luciferase activity (*P* < 0.05), while the transfection with miR-142-5p inhibitor significantly increased the renilla luciferase activity (*P* < 0.05) (Fig. 4E). To assess the effect of miR-142-5p on HFSCs proliferation, the mimic and inhibitor were transfected into HFSCs. In contrast to circ 0020938, the viability of HFSCs transfected with miR-142-5p mimic was significantly increased compared with the control group (*P* < 0.01), while the viability of HFSCs transfected with the inhibitor was significantly decreased than the control group (*P* < 0.01). These results indicated that circ 0020938 could function as a sponge for miR-142-5p (Fig. 4F).

### MiR-142-5p attenuates the inhibitory effect of *DSG4* on HFSCs by targeting *DSG4*

**Fig. 5:**
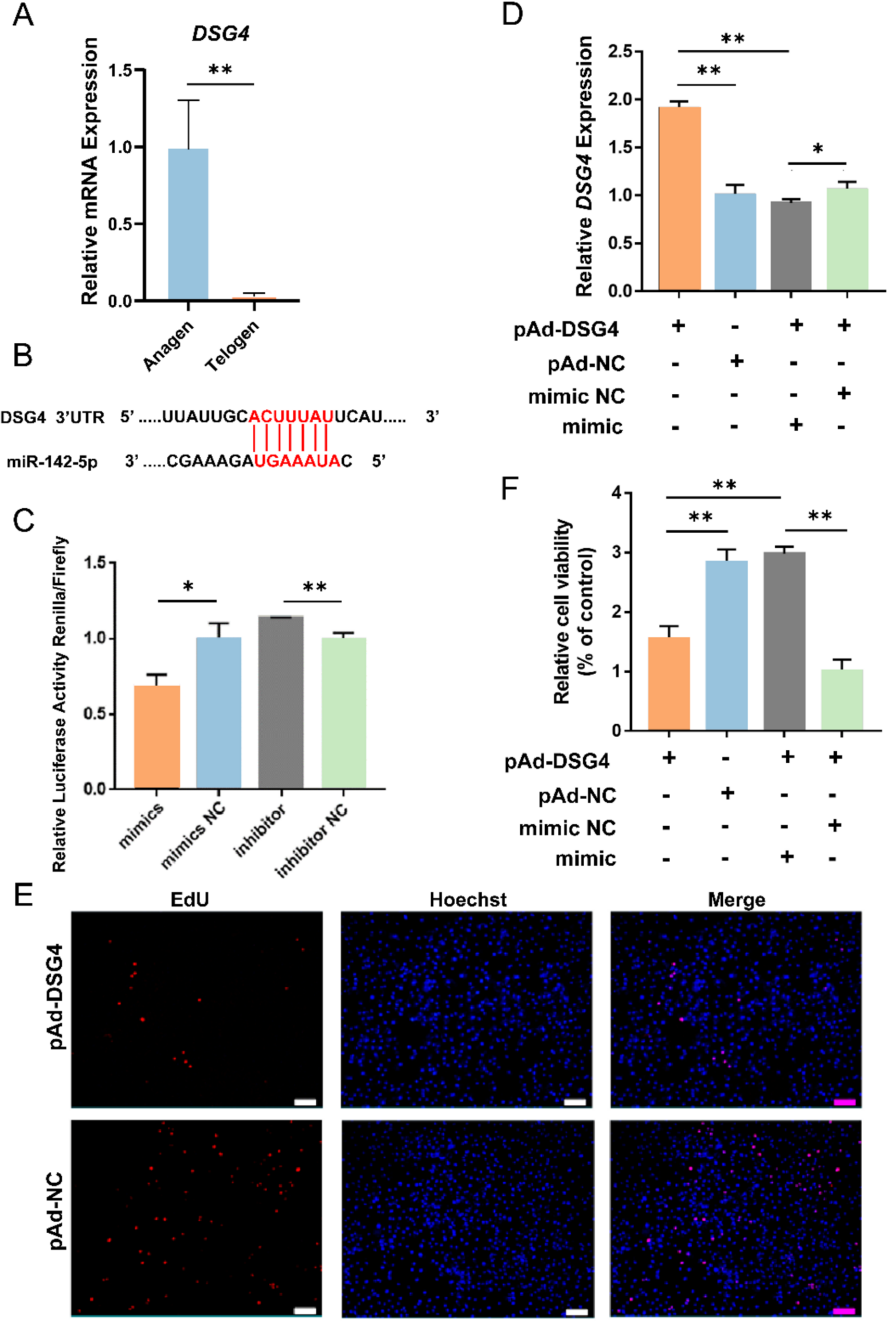
MiR-142-5p attenuates the inhibitory effect of *DSG4* on HFSCs by targeting *DSG4*. **A** The expression level of *DSG4* in the anagen and telogen. **B** Predicted binding sites of *DSG4* and miR-142-5p. **C** The relative luciferase activities were detected in HEK293T cells after co–transfection with psiCHECK-DSG4 3’UTR and mimic or inhibitor. **D** RT-qPCR of *DSG4* expression after transfection with pAd-DSG4, pAd-NC, pAd-DSG4 + miR-142-5p mimic NC, pAd-DSG4 + miR-142-5p mimic. **E** EdU assay detect the effect of *DSG4* on the proliferation of HFSCs. **F** MTT analysis of HFSCs proliferation after transfecting with pAd-DSG4, pAd-NC, pAd-DSG4 + miR-142-5p mimic NC, pAd-DSG4 + miR-142-5p mimic. Data are presented from three independent experiments as mean ± SD, * *P* < 0.05, ** *P* < 0.01. Scale bar, 200 μm

Among the predicted targets, we identified *DSG4*, a core protein essential for normal hair follicle differentiation, which is linked to Monilethrix, a hair loss disorder caused by the mutations in *DSG4* [27, 28]. The RT-qPCR assay results showed that the *DSG4* expression was significantly higher in the anagen stage than that in the telogen stage, contrary to the trend of miR-142-5p expression (*P* < 0.01). Therefore, *DSG4* was selected for further study (Fig. 5A). To determine whether miR-142-5p targets *DSG4*, a dual-luciferase assay was performed (Fig. 5B). Compared to their corresponding controls, the co-transfection of psiCHECK-DSG4 3’UTR with miR-142-5p mimic significantly decreased the renilla luciferase activity (*P* < 0.05), whereas renilla luciferase activity was significantly increased after transfection with inhibitor (*P* < 0.01) (Fig. 5C). Subsequently, the packaged DSG4-overexpressing adenovirus pAd-DSG4 was co-transfected with miR-142-5p mimic into HFSCs, and the result showed that miR-142-5p mimic could significantly inhibit the overexpression effect of pAd-DSG4 compared with the control group (*P* < 0.05) (Fig. 5D). These results all revealed that miR-142-5p was able to target *DSG4*. Immediately, we verified the inhibitory effect of *DSG4* on HFSCs by EdU and MTT assays (Fig. 5E and F). In addition, miR-142-5p mimic was able to restore the inhibitory effect of *DSG4* on HFSCs after the co-transfection of pAd-DSG4 and mimic compared to the control (*P* < 0.01) (Fig. 5F).

### Circ 0020938 inhibits HFSCs proliferation through the MiR-142-5p/*DSG4* axis

**Fig. 6:**
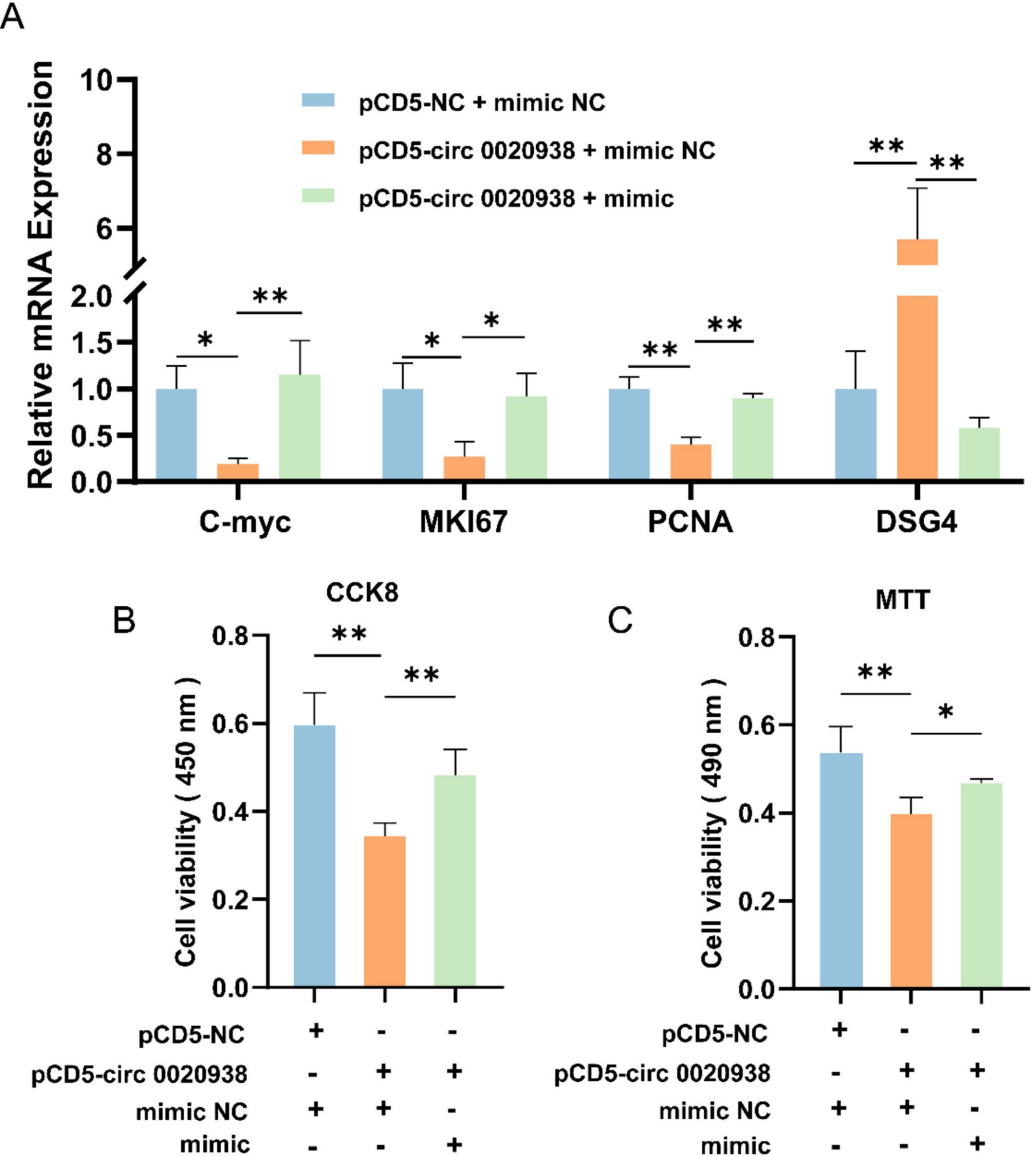
Circ 0020938 attenuated the inhibitory effect of miR-142-5p on *DSG4*. **A** The expression level of proliferation-related genes (*C-myc*, *MKi67*, *PCNA*) was detected by RT-qPCR. **B** CCK-8 assay of HFSCs proliferation after transfecting with pCD5-NC + mimic NC, pCD5-circ 0020938 + mimic NC, or pCD5-circ 0020938 + miR-142-5p mimic. **C** MTT assay detect the viability of HFSCs. Data are presented from three independent experiments as mean ± SD, * *P* < 0.05, ** *P* < 0.01

To further elucidate the relationship between circ 0020938, miR-142-5p, and *DSG4*, we performed a rescue experiment by co-transfecting pCD5-circ 0020938 with miR-142-5p mimic into HFSCs. RT-qPCR analysis showed that compared to the control group, the expression levels of cell proliferation markers *C-myc*, *MKi67*, and *PCNA* were significantly reduced in the pCD5-circ 0020938 + mimic NC group (*P* < 0.05, *P* < 0.01), while the expression of *DSG4* was significantly increased in this group (*P* < 0.01) (Fig. 6A). However, the pCD5-circ 0020938 + mimic group rescued the decrease in the expression levels of cell proliferation markers *C-myc*, *MKi67*, *PCNA* and *DSG4* (*P* < 0.01, *P* < 0.05) (Fig. 6A). In addition, CCK-8 and MTT assays were performed to assess the proliferative capacity of HFSCs under these conditions. Compared to the control group, the viability of HFSCs co-transfected with pCD5-circ 0020938 + mimic NC was significantly reduced (*P* < 0.01). However, the co-transfection of pCD5-circ 0020938 and mimic successfully rescued the reduced cell viability, which significantly increased the proliferative capacity of HFSCs (*P* < 0.05) (Fig. 6B and C). These results indicated that circ 0020938 inhibited HFSCs proliferation through the miR-142-5p/*DSG4* axis.

## Materials and methods

### Data source

The data used in this study were obtained from our previous publication [29] (10.1186/s12864-017-4145-0). The dataset included the samples from three adult female goats at the anagen stage and telogen stage of the hair follicle cycle. The original data were acquired with permission from the authors, and the data files were provided according to the terms outlined in the original publication. Quality control of the raw data was performed following the protocols [7], ensuring the reliability and consistency of the data. In the present study, we further analyzed these data to investigate the changes and potential mechanisms of hair follicle stem cells during different stages of the hair follicle cycle.

### Differential expression, enrichment and CeRNA network analysis of circrnas

The Transcript per Million (TPM) for each circRNA in each sample was calculated by Cuffdiff (v2.1.1) [30]. Differential expression analysis was performed using the DESeq2 R package to compare circRNA expression between the growth and resting stages [25]. GO and KEGG pathway enrichment analyses were performed using Metascape (http://metascape.org).

MiRNAs potentially targeting circ 0020938 were predicted using miRanda and TargetScan software, and circRNA-miRNA gene interaction pairs were constructed. Target genes of miRNAs were predicted using TargetScan intersection with the genes differentially expressed during anagen and telogen. Cytoscape (v3.8.2) [31] software was then used to construct and visualize ceRNA networks.

### Real-time quantitative PCR (RT-qPCR)

RNA was extracted from cells or tissues and reverse-transcribed to cDNA using the PrimeScript™ RT Reagent Kit with gDNA Eraser (Takara, Dalian, China). RT-qPCR was performed on a QuantStudio™ 1 Real-Time PCR Detection System (Thermo Fisher Scientific, USA) using the TB Green™ Premix Ex Taq™ II (Takara, Dalian, China). Gene expression was quantified relative to the endogenous gene expression using the 2^−△△CT^ method. The primers used for RT-qPCR were listed in the supplementary materials.

### Vector construction

The circ 0020938 sequence was amplified from goat cDNA using primers 20,938-pCD-F and 20,938-pCD-R and then cloned into the pCD5 vector named pCD5-circ 0020938. Due to the low transfection efficiency of liposome-based transfection in HFSCs, we performed transfection of pCD5-circ 0020938 using an electroporation system with transfection parameters of: 230 V, 3 ms, and the electrotransfer instrument used was Super Electroporator NEPA21 Type II (NEPA GENE, Japan). Circ 0020938 and DSG4-3′ UTR fragments containing miR-142-5p binding sites were amplified by PCR using goat skin cDNA as template. The amplified fragment was then cloned into the psiCHECK-2 dual luciferase reporter vector (Promega, St. Louis, MO, USA), and the constructs named psiCHECK-circ 0020938 and psiCHECK-DSG4. Transfection of psiCHECK-circ 0020938 and psiCHECK-DSG4 was performed using Lipofectamine 3000 (Invitrogen, Carlsbad, CA, USA) according to the manufacturer ‘s protocol. For overexpression of DSG4, adenoviral plasmids pAd-DSG4 and pAdTrack-GOI plasmids were constructed. MiR-142-5p mimic, inhibitor, and their corresponding negative controls were synthesized by Shanghai GenePharma Co., Ltd. (Shanghai, China). All primers used for vector construction are listed in the supplementary materials.

### HFSCs proliferation assay

HFSCs were primary cells isolated and cultured by our group, and HFSCs were cultured in DMEM/F12 medium containing 10% fetal bovine serum, 1% double antibiotics (Penicillin and Streptomycin) at 37°C, 5% CO_2_. To assess cell viability, the purified HFSCs were seeded into 96-well plates. Once the cell density reached approximately 40–60%, the cells were transfected with pCD5-circ 0020938 or pAd-DSG4. After 24 hours, the medium was replaced with fresh medium. The cells were washed with 100 µL PBS, then 50 µL of 5 mg/mL MTT [3-(4, 5-dimethylthiazol-2-yl)-2, 5-diphenyltetrazolium bromide] (Sigma, St. Louis, MO, USA) reagent was added to each well. The cells were incubated for 4 hours at 37°C in a 5% CO_2_ incubator, and the absorbance was measured at 490 nm. Cell proliferation was also assessed by the Cell Counting Kit-8(CCK-8) assay. After 48 hours of transfection, 10 µL of CCK-8 reagent (Sangon Biotech, Shanghai, China) was added to each well, and the cells were incubated for 1 hour. The absorbance was measured at 450 nm. Cell proliferation was further assessed using the Cell-Light EdU (5-ethynyl-2’-deoxyuridine) Apollo567 in vitro Kit (100T) (RiboBio, Guangzhou, China). After the transfection for 48 h, the medium was changed with the medium containing EdU reagent and incubated with the cells for 6 h. The EdU-positive cells were photographed by IX73 microscope (Olympus, Tokyo, Japan).

### Data analysis

In this study, the data were presented as mean ± SD and analyzed using GraphPad Prism 7.0 software (GraphPad Software, San Diego, CA, USA). Statistical analysis was performed using Student’s *t-test* between two groups. The differences were considered as * *P* < 0.05, ** *P* < 0.01.

## Discussion

Hair follicles, as essential skin appendages, play a critical role in the growth of animal hair and in controlling hair quality and quantity [32]. In normal skin tissues, HFSCs typically remain in a relatively quiescent state [33]. During hair follicle formation, HFSCs differentiate into hair matrix cells, inner and outer root sheath cells, and hair germ cells, all of which are crucial for maintaining hair follicle homeostasis [34].

CircRNAs, a novel class of non-coding RNAs, have been extensively studied in recent years [15, 35]. However, the functional mechanisms of circRNAs in the cyclic regeneration of hair follicles remain largely unexplored. In this study, we constructed an expression profile of circRNAs during the hair follicle cycle. Among the differentially expressed circRNAs, circ 0020938 was identified as being highly expressed during the anagen stage. Surprisingly, overexpression of circ 0020938 inhibited the proliferation of HFSCs. Further analysis revealed that circ 0020938 acts as a ceRNA by sponging miR-142-5p and subsequently regulating the miR-142-5p/*DSG4* axis. MiR-142-5p, a regulatory miRNA, has been reported to play a pivotal role in various skin disorders, such as alopecia areata and melanoma [26]. Meanwhile, DSG4, a desmosomal core protein, has been shown to maintain the structural integrity and the stability of hair follicles [27, 28].

Our results also demonstrated that *DSG4* inhibited the proliferation of HFSCs. During the hair follicle cycle, particularly in the transition from telogen to anagen, HFSCs occupy a central role [36]. At the onset of the anagen stage, HFSCs are activated, transitioning from a quiescent state into a proliferative state [10]. This activation is stimulated by factors in the surrounding microenvironment, primarily via the Wnt/*β-catenin* signaling pathway [37]. Once HFSCs proliferation peaks, some cells begin migrating to the lower portion of the hair follicle, where they differentiate into various follicular cell types [12]. Specifically, HFSCs lose their proliferative capacity and differentiate into inner and outer root sheath cells, providing structural and functional support for hair growth [38]. Over time, hair matrix cells further differentiate into keratinocytes, ultimately forming mature hair [39]. Based on our findings and previous studies, we hypothesize that *DSG4* not only stabilizes hair follicle structure but also regulates the balance between HFSCs proliferation and differentiation during the anagen stage, preventing excessive proliferation and ensuring proper follicular growth and development.

In rescue experiments, the co-transfection of pCD5-circ 0020938 and mimics group displayed significant differences in cell viability and *DSG4* expression levels. Collectively, these findings suggest that circ 0020938 sponges miR-142-5p, attenuating its inhibitory effect on *DSG4*, thereby suppressing HFSCs proliferation. Furthermore, as circRNA research progresses, emerging evidence indicates that the functions of circRNAs extend beyond miRNA sponging. For example, circRNAs can interact with RNA-binding proteins (RBPs) or encode functional peptides [40, 41]. EIcircRNA, a newly identified subclass of circRNAs, has been shown to enhance the transcription of their host genes by interacting with U1 snRNP and RNA polymerase II at the host gene promoter region [42]. CircNEB, a translated circRNA, promotes the proliferation and differentiation of bovine myoblasts in vitro and induces muscle regeneration in vivo [41]. In this study, we successfully constructed the expression profiles of circRNAs during the anagen and telogen stages of hair follicle cycle. Using circ 0020938 as an example, we demonstrated its ability to act as a miRNA sponge to suppress HFSCs proliferation via the miR-142-5p/*DSG4* axis. However, the potential roles of circRNAs in other aspects of the hair follicle cycle remain to be further elucidated.

## Conclusion

This study suggest that circ 0020938 inhibits the proliferation of hair follicle stem cells through the circ 0020938/miR-142-5p/*DSG4* axis. Our findings provide new insights into the molecular regulation of hair follicle stem cells and reveal that circRNAs serve as important regulators of hair follicle biology.

## Electronic supplementary material

Below is the link to the electronic supplementary material.

Supplementary Material 1

Supplementary Material 2

Supplementary Material 3

## Data Availability

The data used in this study were obtained from our previous publication by Wang et al[29] (10.1186/s12864-017-4145-0). The data that support the findings of this study are available from the corresponding author upon reasonable request.

## Abbreviations

HFSC: Hair follicle stem cell
ceRNA: Competing endogenous RNA
ncRNA: Non-coding RNA
GO: Gene ontology
KEGG: Kyoto encyclopedia of genes and genomes
RT-qPCR: Real-time quantitative PCR
TPM: Transcript per million
RBP: RNA-binding protein
