# Supplementary material for: Circ 0020938 inhibits hair follicle stem cells proliferation via the miR-142-5p/DSG4 axis in cashmere goats
Source: BMC Genomics. 2025 May 19;26:505. doi: 10.1186/s12864-025-11642-6 (PMC12090641; doi:10.1186/s12864-025-11642-6)
Supplement: Supplementary file 1 — Supplementary Material 1 [file 12864_2025_11642_MOESM1_ESM.docx]

Table Primers sequence

| Primer | Sequence（5′-3′） |
| --- | --- |
| DSG4-3'UTR-F(Xho I) | *CCGCTCGAG*TGTGTCACTGAAAACGGTCC |
| DSG4-3'UTR-R(Not I) | *ATTTGCGGCCGC*CTTCTCCTGGAAACTGTTTC |
| DSG4-CDS-F(Kpn I) | *GGTACC*ATGGACTGGCTCCTTTTCAGGAA |
| DSG4-CDS-R(Xho I) | *CTCGAG*TTACTGTTGGGAGTAATGTATGTTACTGTACCG |
| q-G-DSG4-F | CGGTGGTGCTGGAAGTAAAC |
| q-G-DSG4-R | GCAGCAAACTTGACCCACTC |
| q-G-ACTIN-F | GGCAACCTCCCCAGTCTCG |
| q-G-ACTIN-R | CCTCAGAAATAGGTCCGAAGC |
| miR-142-5P RT-GSP | gtcgtatccagtgcagggtccgaggtattcgcactggatacgacAGTAGTGC |
| miR-142-5P qRT-F | CGGGCCATAAAGTAGAAAGCAC |
| miR-142-5P qRT-R | CAGTGCAGGGTCCGAGGTAT |
| U6-F | CTCGCTTCGGCAGCACA |
| U6-R | AACGCTTCACGAATTTGCGT |
