# Supplementary material for: Circ 0020938 inhibits hair follicle stem cells proliferation via the miR-142-5p/DSG4 axis in cashmere goats
Source: BMC Genomics. 2025 May 19;26:505. doi: 10.1186/s12864-025-11642-6 (PMC12090641; doi:10.1186/s12864-025-11642-6)
Supplement: Supplementary file 3 — Supplementary Material 3 [file 12864_2025_11642_MOESM3_ESM.pptx]

## Slide 1
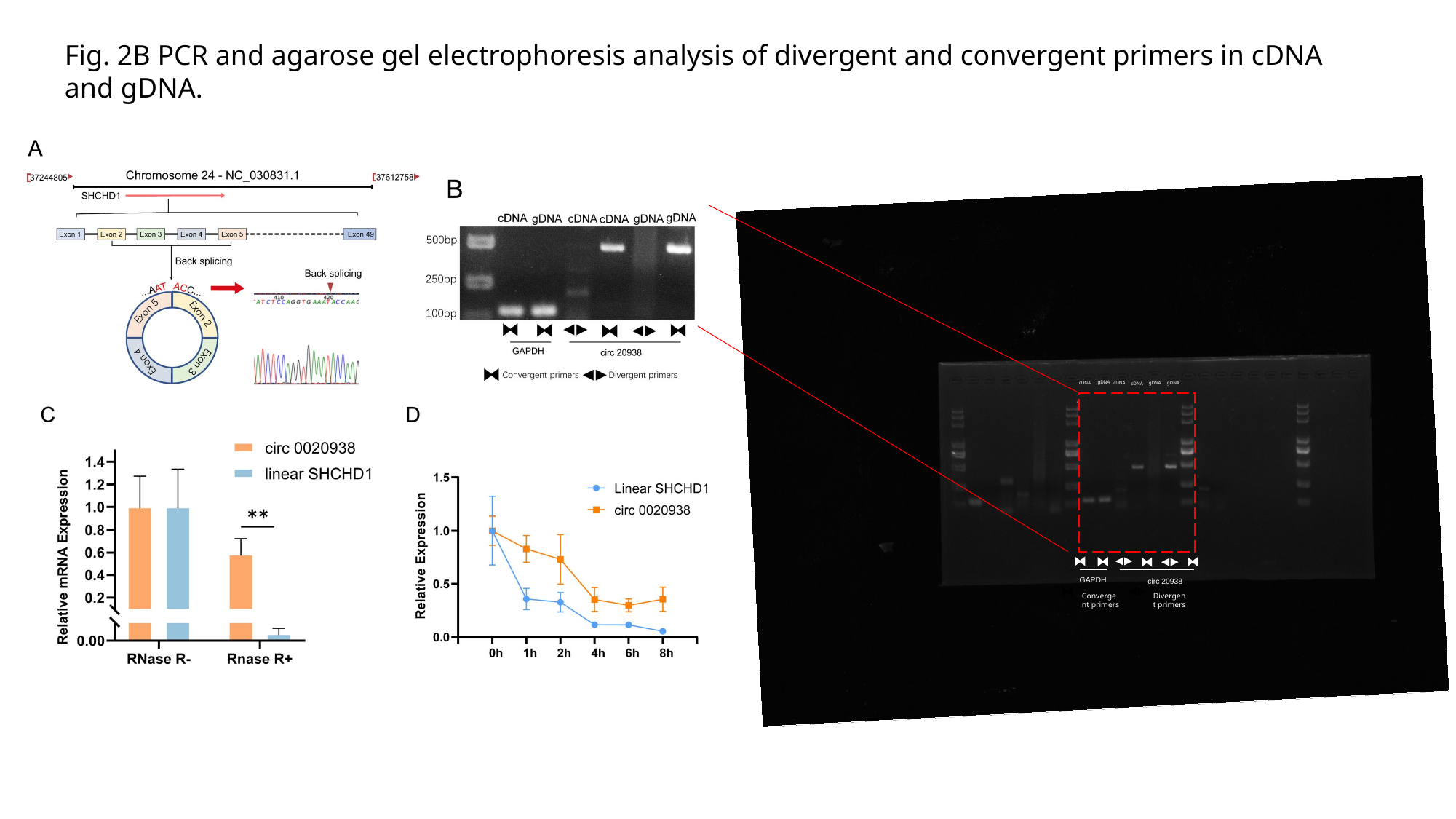

Fig. 2B PCR and agarose gel electrophoresis analysis of divergent and convergent primers in cDNA and gDNA.
gDNA
cDNA
cDNA
gDNA
gDNA
cDNA
GAPDH
circ 20938
Convergent primers
Divergent primers
